# Transcriptomic and metabolomic profiling reveal the p53-dependent benzeneacetic acid attenuation of silica‐induced epithelial–mesenchymal transition in human bronchial epithelial cells

**DOI:** 10.1186/s13578-021-00545-0

**Published:** 2021-02-05

**Authors:** Zhao Ju, Jianlin Shao, Meiling Zhou, Jing Jin, Huiji Pan, Ping Ding, Ruixue Huang

**Affiliations:** 1 Department of Occupational and Environmental Health, Xiangya School of Public Health, Central South University, Changsha, 410078 Hunan China; 2 Zhejiang Provincial Center for Cardiovascular Disease Prevention and Control, Zhejiang Hospital, Hangzhou, Zhejiang China

**Keywords:** p53, Silica, EMT

## Abstract

**Background:**

Silica exposure underlies the development of silicosis, one of the most serious occupational hazards worldwide. We aimed to explore the interaction of the silica-induced epithelial–mesenchymal transition (EMT)-related transcripts with the cellular metabolism regulated by p53.

**Methods:**

We knocked out p53 using CRISPR/Cas9 in the human bronchial epithelial (HBE) cell line. The transcriptomic and metabolomic analyses and integrative omics were conducted using microarrays, GC–MS, and MetaboAnalyst, respectively.

**Results:**

Fifty-two mRNAs showed significantly altered expression in the HBE p53-KO cells post-silica exposure. A total of 42 metabolites were putatively involved in p53-dependent silica-mediated HBE cell dysfunction. Through integrated data analysis, we obtained five significant p53-dependent metabolic pathways including phenylalanine, glyoxylate, dicarboxylate, and linoleic acid metabolism, and the citrate cycle. Through metabolite screening, we further identified that benzeneacetic acid, a key regulation metabolite in the phenylalanine metabolic pathway, attenuated the silica-induced EMT in HBE cells in a p53-dependent manner. Interestingly, despite the extensive p53-related published literature, the clinical translation of these studies remains unsubstantial.

**Conclusions:**

Our study offers new insights into the molecular mechanisms by which epithelial cells respond to silica exposure and provide fresh perspective and direction for future clinical biomarker research and potential clinically sustainable and translatable role of p53.

## Introduction

Silica, a carcinogenic substance, is most commonly associated with a variety of lung diseases including silicosis, lung cancer risk, and the development of autoimmune alteration [1, 2]. Occupational exposure to silica in the mining industry has been observed for almost 150 years [3]. Massive inhalation of silica-based particles leads to lung fibrosis and lung dysfunction. However, at present, the rapid increase in the global population and the corresponding growth in economic development have exponentially increased the number of work environments with silica exposure [4–7]. In the United States (1999–2018), the number of silicosis-related deaths decreased by 40.4 % [8], whereas globally, the numbers increased by 66.0% from 36,000 to 1990 to 60,000 in 2017 [9]. China has reported the highest number (640,000) of silicosis cases over the past three decades [10]. Barnes et al. noted that silica-associated silicosis is a persistent old-world occupational hazard in modern industries [11]. Although the research in the past 20 years has examined the role of silica dust in the development of silicosis, lung injury, and autoimmune diseases, our progress has been limited by the following questions: (1) How does silica exposure lead to varied prognoses and clinical characteristics?; (2) What are the potential mechanisms of lung injury caused by new sources of silica exposure?; (3) How exactly have studies tried to translate to clinic in silica-induced diseases? Our focus in this field at present is to identify evidence that was either previously ignored or missing instead of trying to approach research with mottos such as “100 % preventive” or “zero tolerance in the workplace” [12]. Consequently, a better understanding of the pathogenesis of silica-induced diseases would help discover more precise cellular targets and active signaling pathways to decipher the natural silica-associated lung diseases.

The p53 protein was discovered in 1979 [13] in normal and cancerous cells through four different groups in England, the United States and France simultaneously. Over the past 40 years, p53 has attracted considerable attention and fascination due to its fundamental and important role in gene regulation that safeguards cells against hazardous insults [14]. According to the search results in PubMed (https://pubmed.ncbi.nlm.nih.gov/), more than 100,000 hits were found for literature related to p53 (cumulative of epidemiological and molecular mechanistic research). A typical finding is that p53 function is context-dependent, dependent on the stresses, activating different signaling pathways, and cell states [15]. Indeed, more than 50% of human cancers illustrates p53 mutation, and some of mutant p53 proteins not lose activity but acquire oncogenic function [14]. p53 is also involved in silica-induced silicosis progression. Cheng et al. found that circRNA-012091 promotes lung fibroblast proliferation and migration by regulating a major member of the p53 family, PPP1R13B, in L929 cells [16]. Another study indicated that silica treatment in lung fibroblasts increased p53 expression, enhancing cell migration through activation of the p53/PUMA signaling pathway [17]. Wang et al. considered that the abnormal regulation of p53 by silica might contribute to the development of lung cancer and lung cancer [18]. Although certain studies of p53 involvement in silica-induced diseases had valuable discoveries, there remains much to learn about the roles and regulation of p53 from other new viewpoints. Recently, owing to the development of transcriptomics and metabolomics technology, an increasing number of new p53 functions have been discovered. Wang et al. indicated that a critical metabolite, 20(S)-protopanaxatriol, promoted p53 binding with DNA to form an effective anticancer network through multi-omics detection and analysis [19]. Another study also used integrative omics analysis to obtain a more in-depth understanding of p53-dependent regulation of metabolism, providing a more comprehensive view of its roles in metabolism [20]. Our previous study revealed that through integrative transcriptomics and metabolomics analysis, in response to radiation insult, p53 may dysregulate nitrogen, glutathione, and arachidonic acid metabolism, and glycolysis or gluconeogenesis [21]. This indicates the importance of p53-regulated metabolism in response to environmental toxins and their insults. Hence, we hypothesized that in the silica insulation, lung epithelial cells may be regulated through p53-dependent metabolic pathways, which may be affected by p53 and contribute to the silica-induced lung fibrosis. To verify this, the human bronchial epithelial (HBE) cell line was used to knockout p53 using CRISPR/Cas9. Integrative transcriptomic and metabolomic analyses were performed. Moreover, since p53 research is a hotspot and attracted major attention in scientific community, to further illustrate the p53 research trends globally and provide more information for translating p53 research into clinical application, we also present research and funding trends over the past 10 years.

## Methods and materials

This study has three parts. The first part was the transcriptomics and metabolomics analysis, the second part was the cell mechanism experiment, and the third was the p53 research trends analysis.

### Cell line, construction of p53-knockout (p53-KO) cell line and multi‐omics detection

The HBE cell line was purchased from ATCC (American Type Culture Collection, USA) and stored under the conditions described in our published literature [21, 22]. The P53-KO cells were constructed using the CRISPR/Cas9 technique from Syngentech Ltd. (Beijing, China). The construction details and constructive efficiency were presented in our previous report.

Prior to multi-omics detection, HBE cells were divided into two groups: the p53-wild type (p53-wt) and p53-KO groups. Cells in the two groups were harvested 24 h post 12.5 µg/mL silica treatment. Cells were shipped to OE Biotech Ltd. (Shanghai, China) for further transcriptomic and metabolite analyses. The detection steps of transcriptome sequencing were as follows: cellular total RNA was extracted; the TruSeq Stranded Total RNA LT kit was used to digest RasRNA; confirmed double-stranded cDNA through double-strand synergic reaction system; cDNA was purified; a tail was added to connect sequencing heads; PCR amplification was carried out to construct the RNA library. The GeneChip Fluidics Station 450, GeneChip Hybridization Oven 645, and GeneChip Scanner 3000 7G (Affymetrix, Santa Clara, CA, USA) were used to detect mRNA changes. Two values, fold change and p-value, were used to compare the differential expression status of the mRNAs in the two groups. Fold change was the ratio of the base mean in the p-53KO group to that in the p53-wt group. p < 0.05, and a fold change > 2 were considered to indicate a significant differential expression [21]. Our previous study has reported the effects of a lncRNA–mRNA co-expression network on lung bronchial epithelial cells after radiation exposure [23].

For metabolomics detection, metabolites were extracted from the p53-wt and p53-KO groups after exposure to the same dose of silica and analyzed using gas chromatography-mass spectrometry (GC–MS) [24]. The cell treatments were the same as the ones used for transcriptomics. The GC–MS conditions were as follows: an Agilent 7890B gas chromatography system coupled to an Agilent 5977A mass-selective detector (Agilent Technologies Inc., Santa Clara, CA). The column was an aDb-5 ms capillary column (30 m × 0.25 mm × 0.25 µm: J&W Scientific, Folsom, CA, USA). Carrier gas of high-purity helium with a purity of at least 99.999%. The injection volume was 1 µL with an ample inlet temperature of 260 °C. The initial column chamber temperature was 60 °C, rising to 125 °C at a rate of 8 °C/min, and then to 210 °C at 5 °C/min, followed by heating to 270 °C at 10 °C/min and maintaining at 305 °C for 5 min after increasing the rate of increase to 20 °C/min. The full scan mode (SCAN) was used, with a scanning range of m/z 50–500 [21]. To control the quality, the quality control samples were injected at regular intervals throughout the analytical run.

### Quantitative real‐timepolymerase chain reaction (qRT-PCR)

For qRT-PCR detection, we first extracted RNA using TRIzol (Invitrogen, Carlsbad, CA, USA). Then, the ReverTra Ace qPCR RT Master Mix with gDNARemover (Toyobo, Osaka, Japan) was used to reverse-transcribec DNA based on the manufacturer’s instructions. qRT-PCR was carried out in our laboratory using the Super Real PreMix Plus (SYBR Green) kit (Tiangen Biotech, Beijing, China) on the CFX96TM Real-Time system (Bio-Rad Laboratories, Hercules, CA, USA), to validate the changes and trends observed in the microarray analysis. The comparative Ct(2 −ΔΔCt) method was used to calculate relative fold differences in mRNA expression between HBE p53-KOand HBE p53-wt cells. The calculated formula was 2^−ΔΔCt^ = ΔCt(p53-KO)/ΔCt(p53-wt)(2^−ΔΔCt ^≥ 2 indicated high expression).

### Bioinformatic and integrative analyses

We performed bioinformatic analysis and integrative analysis on the results of transcriptomic and metabolomics profiling. Bioinformatic analysis included heat map construction, volcano map construction, GO (Gene Ontology) analysis, and KEGG (Kyoto Encyclopedia of Genes and Genomes) pathway analysis. GO analysis mainly comprises molecular functions, cellular components, and biological processes, which are available online http://geneontology.org/. The KEGG pathway analysis is a collection of manually-drawn pathway maps representing our knowledge of the molecular interaction, reaction, and relation networks for metabolism, genetic information processing, cellular processing, and so on available online https://www.kegg.jp/kegg/pathway.html.  Integrated transcriptomic and metabolomic analyses were conducted using MetaboAnalyst (https://www.metaboanalyst.ca/MetaboAnalyst/home.xhtml). This software performed integrative analysis through a completely revamped web interface, with three new modules (MS Peaks to Pathways, Biomarker Meta-analysis, and Network Explorer), together with a companion R package (MetaboAnalystR) [25, 26]. Gene lists and metabolite lists were uploaded with fold changes > 2 and p < 0.05. The algorithm selection was a hypergeometric test, the topology measure was degree centrality, and the integration method combined queries.

### Cell experiments

HBE cells were used to assess the IL4I1, p53, E-Cadherin mRNA levels changes following different metabolite treatments through qRT-PCR detection. Differential benzeneacetic acid treatments (50 µM, 100 µM, and 150 µM) and the effects on cell survival rate via the CCK-8 method using the relative CCK-8 assay kit, cell cycle changes after treatment with or without benzeneacetic acid and the biomarkers of epithelial–mesenchymal transition (EMT) changes with or without benzeneacetic acid treatment. These cell experiments were conducted to compare the differential changes and functions between p53-wt and p53-KO cells.

The CCK-8 assay was conducted as previously described [27]. Briefly, after adding 10 µL CCK-8 reagent, the cells were mixed evenly and kept in an incubator for 1 h. The absorbance (A) at 450 nm was measured using an enzyme-linked immunosorbent assay reader, and the growth curve was plotted. Three wells were set up in each group [27].

Then, flow cytometry was used for the detection of the cell cycle. The two groups of cells were fixed with 70 % ethanol and then placed overnight in the refrigerator at 4 °C. The cells were washed twice with pre-cooled PBS, and PI dyeing solution (20×) and RNase A (50×) were added to each well with cells. After incubation at 37 °C in the dark for 0.5 h, the cell cycle was detected using flow cytometry.

WB detection was conducted as previously reported [22, 28, 29]. In this study, antibodies against p53, E-cadherin, N-cadherin, and Vimentin were purchased from Cell Signaling Technology, Inc.(Danvers, MA, USA) and Abcam (Cambridge, UK). Images were captured and assessed using the ChemiDocXRS + system (Bio-Rad Laboratories). Protein expression was quantified using Image Lab software (Bio-Rad Laboratories). At least three independent replicates were analyzed for each sample.

### p53 research trends analysis

To draw a global view of p53 research trends, we first used the keyword “p53”to search for p53-related funds over the past decade in the online National Natural Science Foundation of China (NSFC; http://www.nsfc.gov.cn/), and the National Institute of Health in the USA (https://www.nih.gov/). We then used ArcGIS software to illustrate the funding number in the differential province in China. Then, p53-related publications over the decade were searched in the PubMed database, and the number of publications in China and the USA were also compared. Finally, we searched the p53-related invention patents from the Chinese, USA, and European patent databases in our university (Jove Eye; http://lib.joveeye.com).

### Statistical method

All data from the statistical analysis are presented as means ± standard deviation (SD). The data were analyzed using SPSS 20.0, GraphPad Prism 8.0 (Graph-Pad Software Inc., San Diego, USA) and R 3.2.5 (DevelopmentCore Team, Vienna, Austria). The independent sample mean t-test was used for comparison between two groups. mRNA expression levels were compared using the two-tailed Student’s t-test. *p* < 0.05 indicated a significant difference.

## Results

### Deficiency of p53 significantly alters mRNA expression following silica stimulus

**Fig. 1:**
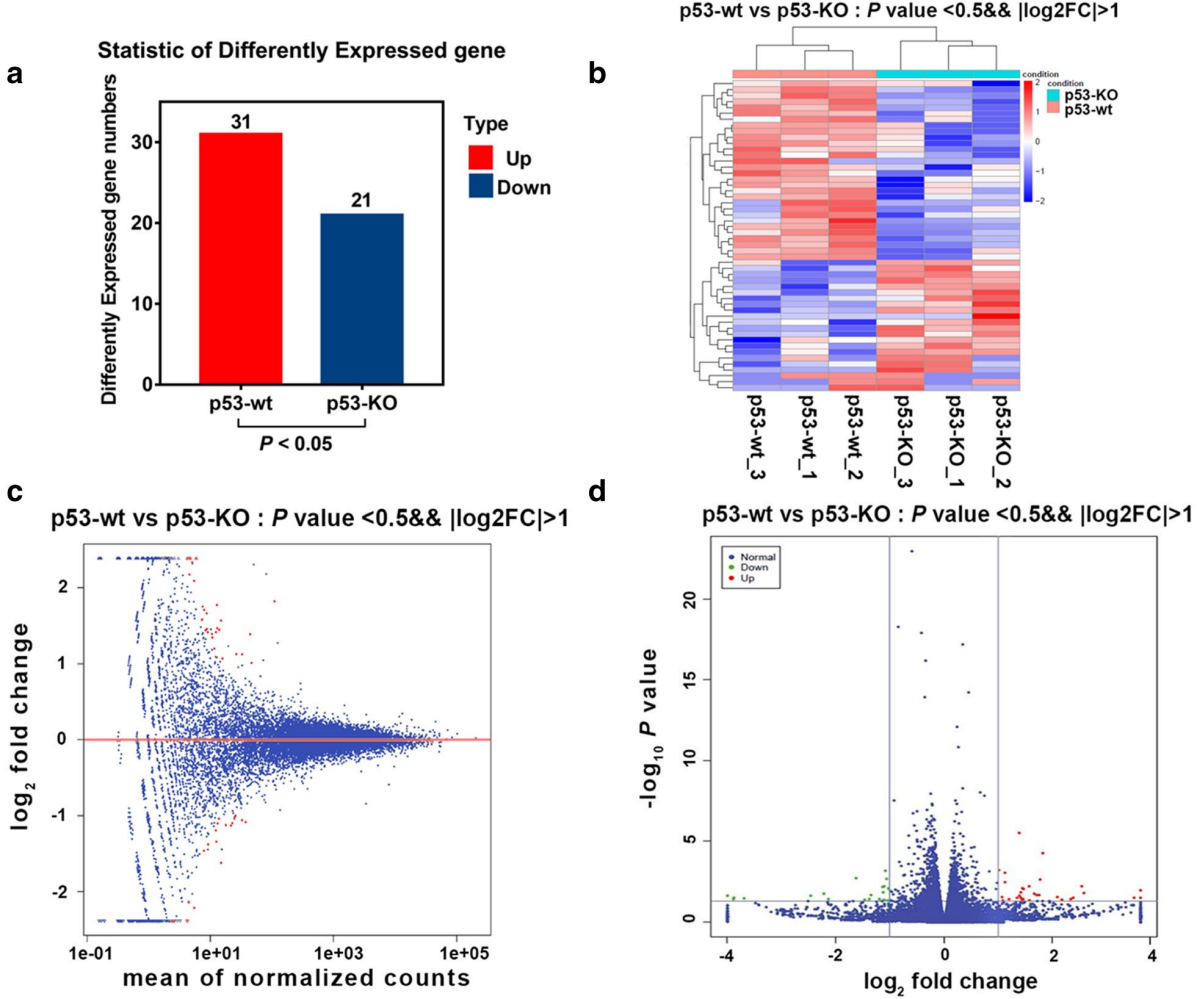
Significant differentially expressed genes between p53-wt and p53-KO cells post silica stimuli. HBE cells with or without p53 expression mediated by CRISPR/Cas9 knockout technology were divided into two groups, p53-wt and p53-KO. Cells were harvested at 24 h, followed by treatment with 12.5 µg/cm^2^ silica. **a **A total of 52 genes were significantly altered, of which 31 genes were significantly upregulated in the p53-KO group compared with that of the p53-wt group post silica treatment (*p* < 0.05). Twenty-one genes were significantly downregulated in the p53-KO group compared with that of the p53-wt group post silica treatment (*p* < 0.05). **b** A heatmap illustrating the differentially expressed mRNAs in HBE cells with (p53-wt) or without CRISPR/Cas9-mediated *p53-*knockout (p53-wt) after silica exposure. Red represents upregulated and purple represents downregulated mRNAs. **c** A MA map illustrating the distribution of standardized genes. Red represents significant differentially expressed genes whereas blue represent genes with no significant changes in expression. **d** A volcano map illustrating the differentially expressed mRNAs in HBE cells with (p53-KO) or without CRISPR/Cas9-mediated *p53-*knockout (p53-wt) after silica. Red represents upregulated mRNA, green represents downregulated mRNA and blue represents the mRNA with no changes in expression

The verification of p53-knockout efficiency using CRISPR/Cas9 was examined with WB. The p53-knockout result has been reported in our previous study. In this study, we continued to ascertain whether p53 deficiency influenced the mRNA alterations following silica (12.5 µg/L) treatment in the p53-wt and p53-KO HBE cells. At 24 h post silica treatment, compared with the p53-wt group, mRNAs showed significant alterations in the p53-KO group. The expression levels of a total of 52 mRNAs changed significantly (*p* < 0.05), of which 31 mRNAs were upregulated and 21 were downregulated in the p53-KO group (Fig. 1a). We harvested cells for transcriptomic detection based on our findings from our pilot study that suggested that cell damage at this timepoint could significantly induce EMT [29–31]. The details of the changes in mRNA expression are listed in Table 1. It can be seen that ANKRD22, GALNT16, LGR5, ITGA9, and ELMOD1 were the five most strongly upregulated mRNAs, with fold changes of approximately 11, 6, 5.8, 5.2, and 5, respectively. Meanwhile, SLC9B1, GOLGA8Q, FAM19A5, LAIR2, and PLA2G2C were the five most strongly downregulated mRNAs, with downregulation fold changes of 0.05, 0.06, 0.06, 0.07, and 0.18, respectively. Heatmap and volcano map assays further illustrated that compared with p53-wt cells, the number of the upregulated mRNAs was significantly higher than that of the downregulated mRNAs in the p53-KO cells (Fig. 1b–d). These results demonstrated that p53 deficiency led to changes in mRNA expression upon exposure to silica, suggesting that mRNA expression may be directly or indirectly regulated by p53.

**Table 1:** Top 20 differential expressed genes/mRNAs in the p53-wt vs. p53-KO HBE cells post 12.5 µg/mL silica

Upregulated mRNA				Downregulated mRNA			
Gene-ID	Fold change	Log2 (Fold)	*p value*	Gene-ID	Fold change	Log2 (Fold)	*p value*
ANKRD22	11.33825	3.503126	0.031888	SLC9B1	0.062922	− 3.99028	0.023555
GALNT16	5.9697	2.577658	0.015915	GOLGA8Q	0.067677	− 3.88519	0.040364
LGR5	5.805771	2.537488	0.00641	FAM19A5	0.068239	− 3.87326	0.033308
ITGA9	5.201689	2.37898	0.033195	LAIR2	0.07763	− 3.68725	0.035158
ELMOD1	5.045316	2.334945	0.038847	PLA2G2C	0.182084	− 2.45733	0.024876
GPT	4.512969	2.174077	0.042918	TNFRSF10C	0.215358	− 2.21519	0.018619
SCG3	4.258003	2.090177	0.028393	NEU4	0.227828	− 2.13399	0.040534
DEFA5	3.533064	1.82092	5.51E-05	VAV1	0.325185	− 1.62067	0.002011
DEFA6	3.417341	1.772874	0.002371	SHC2	0.36299	− 1.462	0.044092
CRYBB2	3.367854	1.751829	0.022507	LOC107985524	0.384776	− 1.37791	0.040135
JAKMIP2	3.267922	1.708373	0.022156	DLX2	0.394143	− 1.34321	0.022467
ZNF385B	3.169598	1.6643	0.018243	FNTB	0.437081	− 1.19403	0.039543
PRSS2	2.980291	1.575453	0.046668	COL8A2	0.456718	− 1.13063	0.006895
RNF103-CHMP3	2.964665	1.567869	0.00646	PSG8	0.458618	− 1.12464	0.015305
CHRNA1	2.764834	1.467193	0.009205	SLC35F3	0.465274	− 1.10385	0.041656
LRRC46	2.747	1.457857	0.04034	AS3MT	0.466297	− 1.10068	0.006206
ADCY4	2.725217	1.446371	0.008368	TREM2	0.47154	− 1.08455	0.000655
LIX1	2.71129	1.438979	0.041953	KCNMA1	0.479557	− 1.06023	0.002141
KCND3	2.666753	1.415084	0.015511	NANOS3	0.486488	− 1.03952	0.046132
SLC24A3	2.665332	1.414315	0.033314	SLC45A1	0.491734	− 1.35907	0.008291

*p* < 0.05 represents significantly changed

**Fig. 2:**
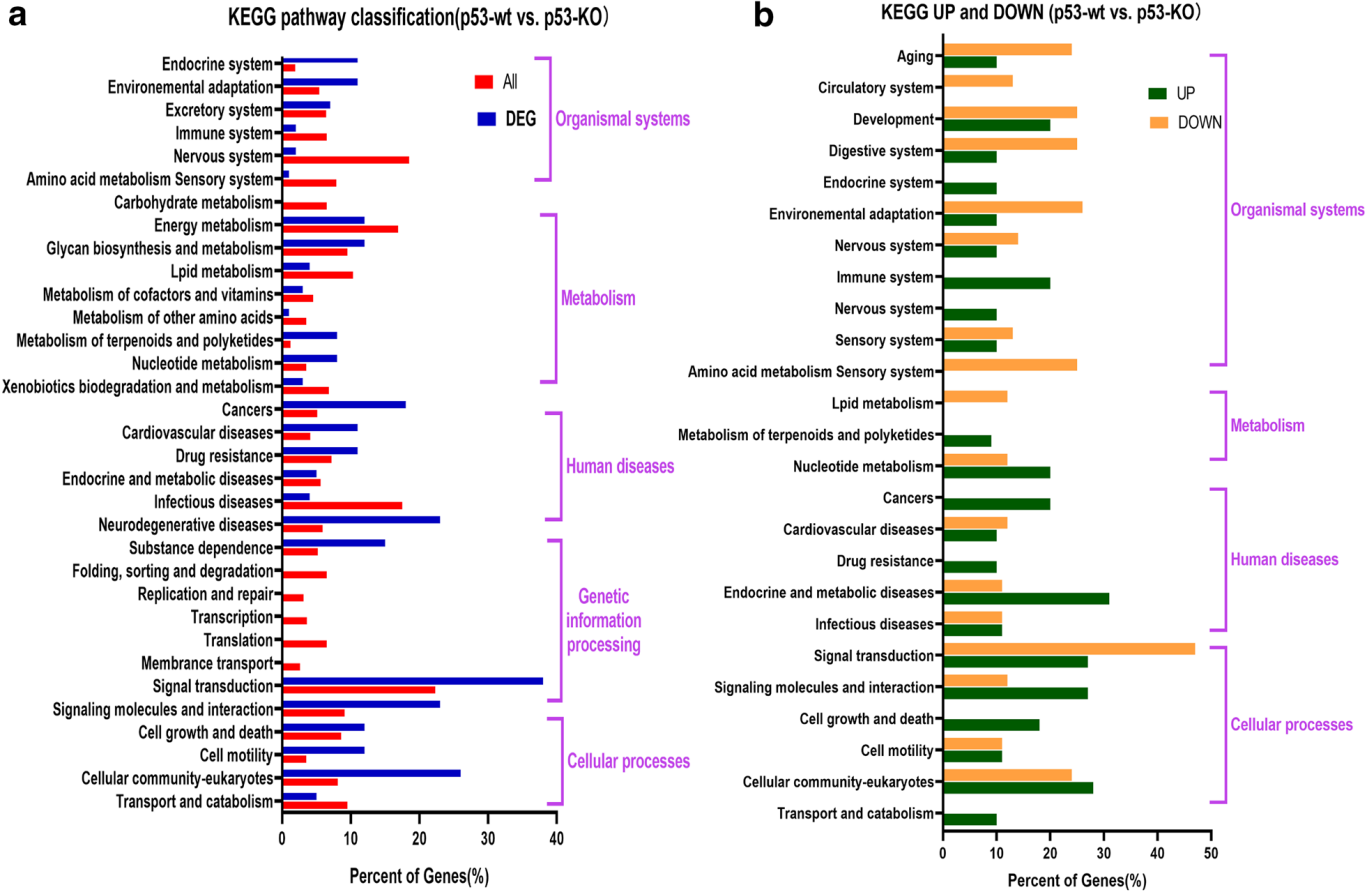
GO and KEGG pathway analyses of significant differentially expressed mRNAs in HBE cells with or without CRISPR/Cas9-mediated *p53-*knockout. **a** Enrichment of top 30 mRNAs with differential expression, involved in various biological processes, cellular components, and molecular junctions. **b** KEGG pathway classification of differentially expressed mRNAs in p53-wt vs. p53-KO. **c** KEGG pathway classification of upregulated and downregulated mRNAs with significant differential expression in p53-wt vs. p53-KO. **d** Verification of selected randomly relative mRNA expression in the p53-KO group compared with that of the p53-wt group

To predict the differentially altered mRNA functions, we performed GO analysis on the top 30 differentially expressed mRNAs. As shown in Additional file 1: Fig. S1, the altered mRNAs were related to a large variety of cell structures and functions, including molecular junctions such as the nucleus, calcium ion binding, cellular components including the extracellular region and plasma membrane, biological processes including antimicrobial humoral response, and regulation of cell proliferation. To compare the signaling pathways involved between all differentially altered mRNAs (*p* < 0.05), we performed KEGG pathway analysis. The mRNAs with a significantly altered expression (Fig. 2a) were mainly involved in downstream signaling pathways, environmental adaption, nervous system, energy metabolism, lipid metabolism, glycan biosynthesis and metabolism, xenobiotics biodegradation, and metabolism. Furthermore, compared to the significantly downregulated mRNAs, the upregulated mRNAs were involved in the signaling pathway of the immune system, nucleotide metabolism, cancers, endocrine and metabolic diseases, and transport and catabolism. These data indicate that the deficiency of p53 significantly alters the expression of a large number of mRNAs and that these mRNAs may participate in cellular function in response to silica. Figure 2b, Additional file 2: Fig. S2 show the consistency of the microarray chip using five upregulated and five downregulated mRNAs. Our results were consistent with the microarray chip. This demonstrated that the microarray chip results can be used for downstream analyses and provide baseline information for cell experimentation.

### Deficiency of p53 significantly alters silica-mediated metabolites

**Fig. 3:**
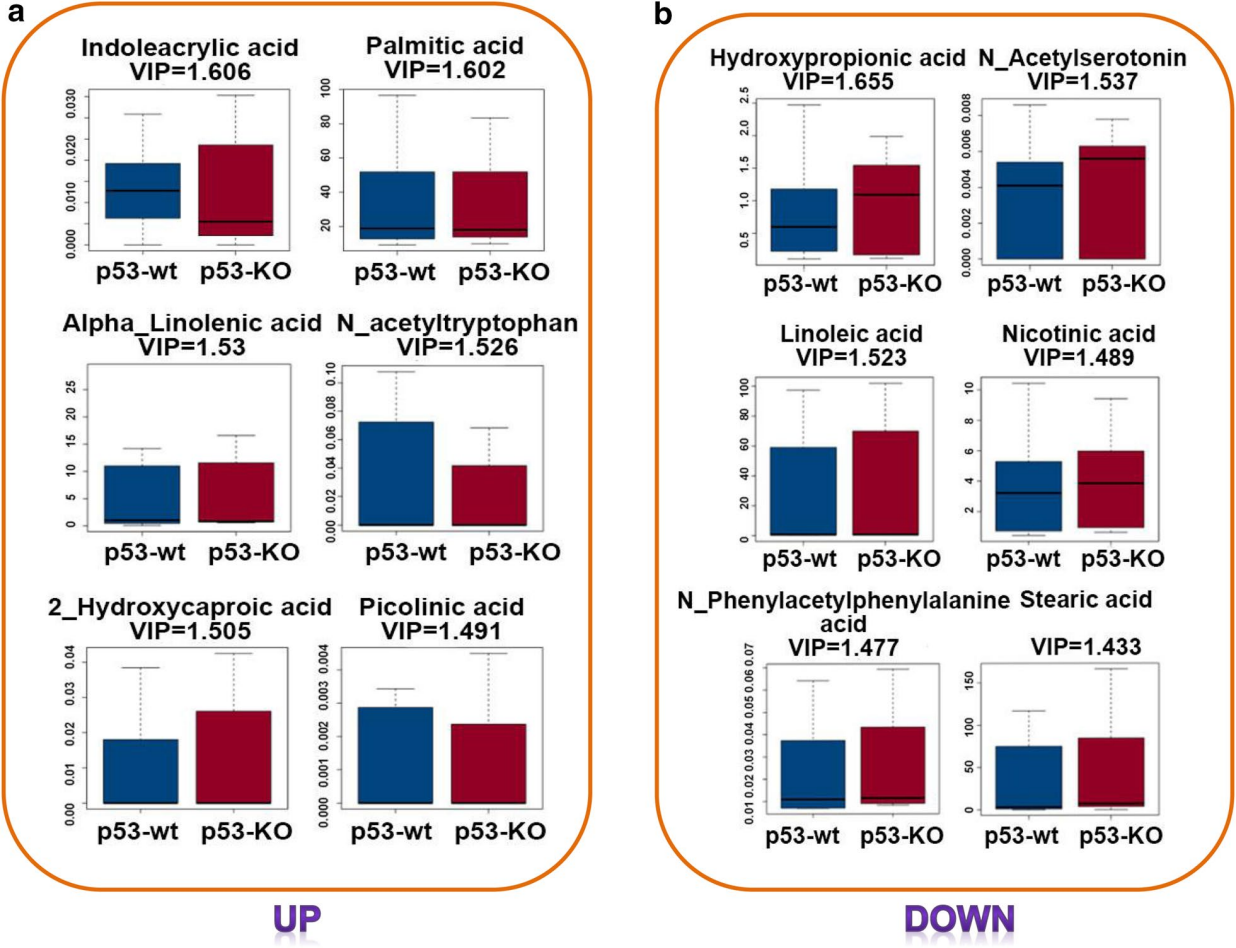
Top six upregulated or downregulated metabolites in the p53-KO group compared with that of the p53-wt group post silica exposure. **a** Top six upregulated metabolites in the p53-KO group compared with that of the p53-wt group post silica exposure. **b** Top six downregulated metabolites in the p53-KO group compared with that of the p53-wt group post silica exposure. VIP means variable importance in the projection

Metabolic adaption is an adaptive change occurring during the cells in response to silica under the p53-existing or deficiency status. The final goal of this adaptive change is to achieve a new homeostatic status and maximize the opportunity for cell survival [32]. In total, 42 metabolites changed significantly in HBE p53-KO cells compared with p53-wt cells (*p* < 0.05), of which 22 were upregulated and 20 were downregulated. Table 2 lists the 18 most altered metabolites. Among the top 18 differential metabolites, linoleic acid, butyric acid, alpha linolenic acid, stearic acid, and arachidonic acid were the five most strongly upregulated metabolites, with fold changes of approximately 91, 17, 16, 13, and 8, respectively. Oxoglutaric acid, beta_alanine, fumaric acid, 4_hydroxyphenylpyruvic acid, and l_Thyronine were the five most strongly downregulated metabolites, with fold changes of approximately 0.05, 0.08, 0.1, 0.13, and 0.16, respectively. Metabolomics profiling demonstrated that p53 deficiency resulted in the dysregulation of multiple metabolites, which may result in changes in related metabolic pathways. Variable important in projection (VIP) analysis of significantly changed metabolites was conducted. The larger the VIP, the greater the contribution of this variable to the grouping. Using 1.00 as the VIP cutoff score, 18 upregulated and 18 downregulated metabolites were identified as being potentially closely associated with p53 deficiency, of which the six upregulated and six downregulated metabolites are presented in Fig. 3a, b.

**Table 2:** Top 18 differential metabolites in the p53-wt vs. p53-KO HBE cells post 12.5 µg/mL silica

Upregulated metabolites				Downregulated metabolites			
Gene-ID	Fold change	Log2 (Fold)	VIP score	Gene-ID	Fold change	Log2 (Fold)	VIP score
Linoleic acid	91.30556568	6.5126309	1.522815	Oxoglutaric acid	0.054212046	− 4.205242732	1.153971
Butyric acid	17.21165834	4.105314202	1.239433	Beta_Alanine	0.083038327	− 3.590078819	1.266837
Alpha_Linolenic acid	16.48137866	4.042765023	1.529557	Fumaric acid	0.106235462	− 3.234662673	1.066492
Stearic acid	13.95122891	3.802320304	1.432926	4_Hydroxyphenylpyruvic acid	0.138183866	− 2.855338915	1.262136
Arachidonic acid	8.151991288	3.02715251	1.307153	l_Thyronine	0.160574413	− 2.638686077	1.33295
Docosahexaenoic acid DHA	7.888603473	2.979769921	1.409017	Pyroglutamic acid	0.183156185	− 2.448853673	1.0547
Valeric acid	6.513822845	2.703504484	1.326264	p_Hydroxyphenylacetic acid	0.18381705	− 2.443657506	1.293357
Formic acid	5.821979245	2.541509696	1.244691	Hydroxyphenyllactic acid	0.188740975	− 2.405520436	1.228796
11_*cis*_Eicosenoic acid	4.970626572	2.313427722	1.428579	3_Nitrotyrosine	0.214676537	− 2.219763575	1.294143
Indoleacrylic acid	4.329224483	2.11410861	1.606161	Benzeneacetic acid	0.221848007	− 2.1723565	1.111947
Pentadecanoic acid	3.848793752	1.944406363	1.180618	Hydroxypropionic acid	0.245830401	− 2.024264755	1.654862
Palmitic acid	3.779422024	1.918165624	1.601808	Glyceric acid	0.258762543	− 1.950299297	1.025171
*N*_Phenylacetylphenylalanine	3.737134456	1.90193247	1.476966	l_Alanine	0.259966065	− 1.943604783	1.098831
3,4_Dihydroxyhydrocinnamic acid	3.660604188	1.872081787	1.231095	*Cis* and *trans*_Cinnamic acid	0.271785878	− 1.879457598	1.155961
Docosapentaenoic acid 22n_6	2.176457553	1.121981884	1.026765	Phenylacetic acid	0.28363417	− 1.817896749	1.418577
Isobutyric acid	1.771921197	0.825314444	1.012767	Nicotinic acid	0.284218303	− 1.814928632	1.497762
Heptadecanoic acid	1.599045316	0.677210825	1.189061	*N*_Acetylserotonin	0.28649635	− 1.803411334	1.53686
Docosapentaenoic acid DPA	1.480405616	0.565992513	1.072228	Suberic acid	0.303340107	− 1.720991836	1.305605

VIP: Variable important in projection, The larger the VIP, the greater the contribution of this variable to the grouping; Log2 (FC): the ratio of the average expression amount of metabolites in the two groups of samples. A positive value indicates up regulation and a negative value indicates down regulation

**Fig. 4:**
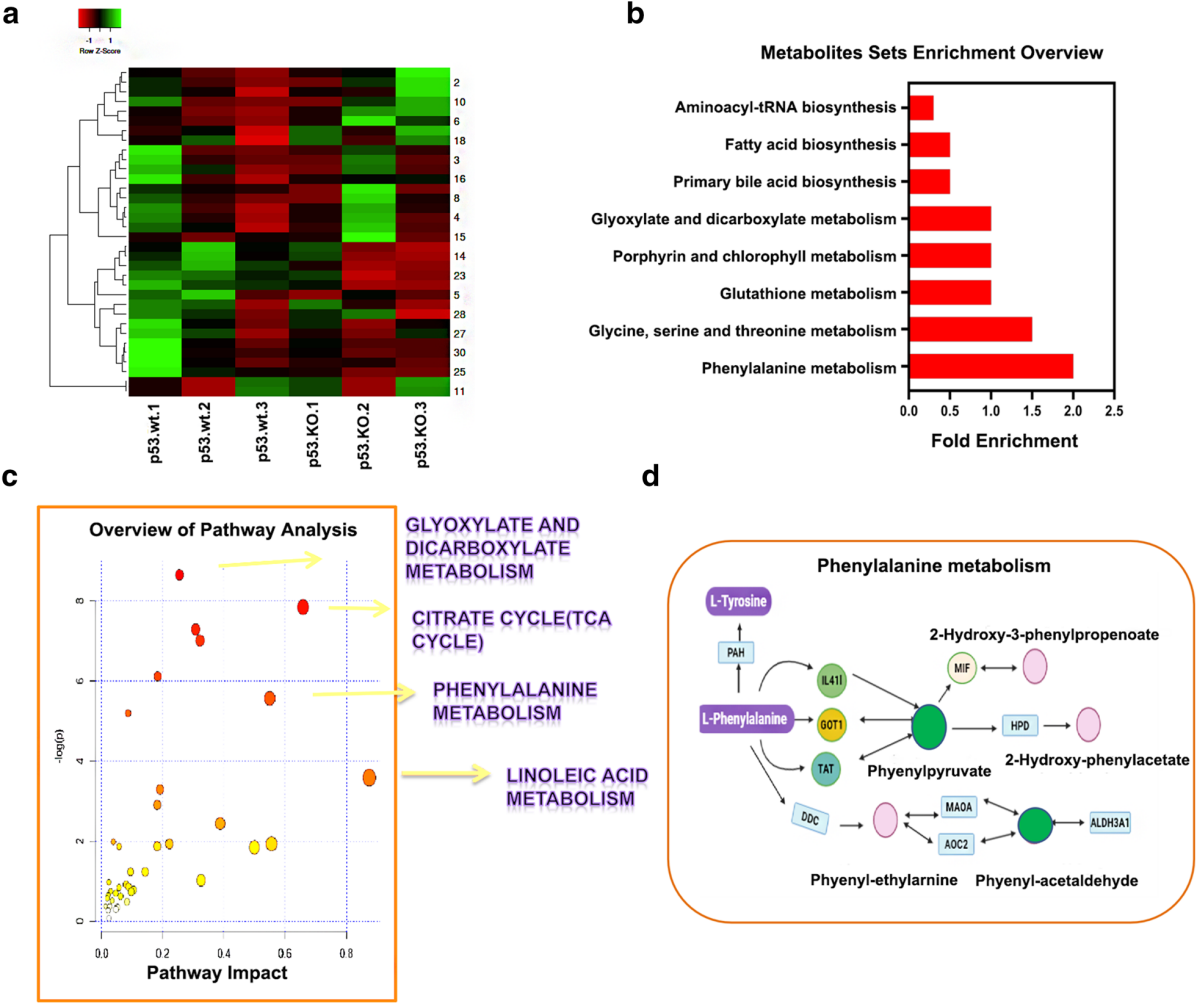
Bioinformatic analysis of metabolomics. **a** A heatmap was used to visually represent upregulated and downregulated metabolites. **b** KEGG analysis illustrates the enrichment pathway for the significantly differential expressed metabolites. **c** Integrated transcriptomic and metabolomic analyses of p53-dependent metabolic pathways. **d** Details of phenylalanine metabolism. Pink represents slightly upregulated metabolites or genes, green represents significantly downregulated metabolites or genes, blue represents no significant changes

The heatmap visually represents upregulated and downregulated metabolites. As shown in Fig. 4b, compared with the p53-wt group, metabolites were significantly upregulated in the p53 knockout group post-silica. KEGG enrichment pathway analysis showed that the significantly changed metabolites were enriched in phenylalanine metabolism, Glycine, serine, threonine, glutathione, porphyrin, chlorophyllglyoxylate, and dicarboxylate metabolism (Fig. 4b). Metabolomic data confirmed that p53 has a wide range of regulatory effects on intracellular metabolism via these metabolic pathways in response to silica.

### Integrated transcriptomic and metabolomic analyses of p53-dependent metabolic pathways

As our purpose of this study aimed at characterizing the transcriptomics and metabolomics changes occurring in HBE cells under silica exposure to compare p53 interactions and connections, we performed multi-omics analysis [33]. Notably, multi-omics can discover significantly perturbed pathways at both the metabolic and transcriptional levels, thereby providing a potential biomarker of silica-induced EMT and a basis for further investigation of the underlying molecular mechanisms [33]. Online MetaboAnalyst 4.0, using the PrimeFaces library (v7.0) based on the JavaServer Faces Technology, offering an efficient pipeline to support high-throughput global metabolomics in the open-source R environment [25] was utilized to perform our multi-omics in this study (https://www.metaboanalyst.ca/home.xhtml).

**Fig. 5:**
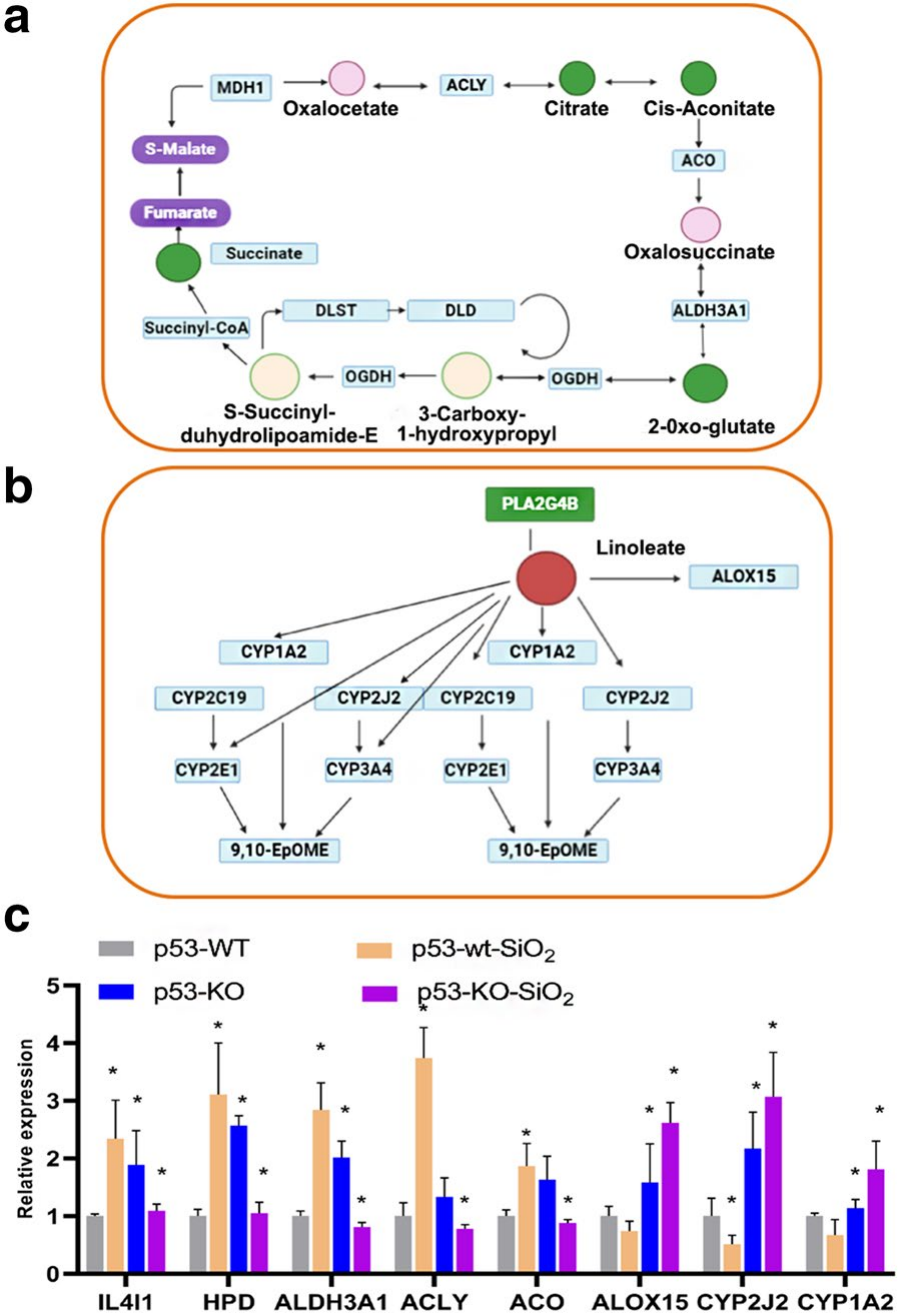
Integrated transcriptomic and metabolomic analyses of p53-dependent metabolic pathways. **a** Details of the citrate cycle (TCA). Pink represents slightly upregulated metabolites or genes, green represents significantly downregulated metabolites or genes, blue represents insignificant changes. **b** Details of linoleic acid metabolism. Red represents significantly upregulated metabolites or genes, green represents significantly downregulated metabolites or genes, blue represents significant changes. **c** IL4I1, HPD, ALDH3A1, ACLY, ACO, ALOX15, CYP2J2, and CYP1A2 expression levels were determined using qRT-PCR in HBE cells of p53-wt, p53-wt treated with silica (p53-wt-SiO_2_), p53-KO, and p53-KO treated with silica (p53-KO-SiO_2_), respectively. Data are presented as means ± SD from three independent experiments; *p < 0.05 between different groups

The mRNA enrichment and metabolic pathway analyses revealed 35 significantly altered pathways at both the metabolomic and mRNA expression levels in HBE p53-KO cells, where these pathways are involved in glyoxylate and dicarboxylate metabolism, citrate cycle (TCA), arginine biosynthesis, butanoate metabolism, alanine, aspartate, and glutamate metabolism, and phenylalanine metabolism (Table 3). Figure 5b shows that there are four metabolic pathways, phenylalanine, glyoxylate, dicarboxylate, and linoleic acid metabolism and TCA had *p*-values < 0.05 and impact coefficients > 0.2, indicating that, in response to silica insult, these pathways may be dysregulated by p53. Figure 4d illustrates the details of the phenylalanine metabolic pathway, indicating that phyenylpyruvate and phenyl-acetaldehyde levels and TAT expression were decreased and 2-hydroxy-3-phenylpropanoate and 2-hydroxy-phenylpropanoate levels were increased. Figure 5a illustrates the details of the TCA pathway, indicating that the citrate, *cis*-aconitate, 2-oxo-gluate, and succinate were decreased and oxaloacetate and oxalosuccinate levels were increased. Figure 5b illustrates the details of the linoleic acid metabolic pathway, indicating that the linoleate was significantly increased, but PLA2G4B was decreased. These data indicate that the changes seen in mRNA expression levels may be related to p53-dependent alteration of metabolic pathways.

**Table 3:** Integrative metabolic pathways analysis on results obtained from combined metabolomics and gene expression

Pathway name	Match status	*p*	Impact
Glyoxylate and dicarboxylate metabolism	10/92	0.000174	0.25455
Citrate cycle (TCA cycle)	8/142	0.000391	0.65854
Arginine biosynthesis	6/76	0.000679	0.30769
Butanoate metabolism	6/77	0.000899	0.32143
Alanine, aspartate and glutamate metabolism	6/82	0.002202	0.18333
Phenylalanine metabolism	5/50	0.003821	0.55
Biosynthesis of unsaturated fatty acids	6/106	0.005523	0.086957
Linoleic acid metabolism	5/64	0.027727	0.875
Propanoate metabolism	6/115	0.037239	0.19149
Glutathione metabolism	5/70	0.054837	0.18182
Glycine, serine and threonine metabolism	4/44	0.08738	0.38806
Fatty acid biosynthesis	4/70	0.13803	0.039062
d-Glutamine and d-glutamate metabolism	5/132	0.14551	0.22222
Synthesis and degradation of ketone bodies	4/79	0.14551	0.55556
Pyruvate metabolism	4/92	0.15422	0.18182
Tyrosine metabolism	4/114	0.15534	0.057471
Phenylalanine, tyrosine and tryptophan biosynthesis	3/57	0.15888	0.5
Alpha-Linolenic acid metabolism	4/117	0.29307	0.095238
Mucin type O-glycan biosynthesis	3/74	0.29307	0.14286
Arachidonic acid metabolism	4/148	0.36168	0.325
Tryptophan metabolism	3/90	0.37875	0.024096
Valine, leucine and isoleucine degradation	2/33	0.40123	0.08046
Glycerolipid metabolism	3/94	0.42493	0.088235
Terpenoid backbone biosynthesis	3/94	0.43402	0.057143
Ether lipid metabolism	3/98	0.46045	0.10526
Fatty acid degradation	2/43	0.47685	0.029703
Nicotinate and nicotinamide metabolism	3/124	0.48567	0.097561
Beta-Alanine metabolism	2/69	0.50185	0.046512
Pentose phosphate pathway	2/73	0.52518	0.021739
Lysine degradation	1/12	0.54015	0.0625
Porphyrin and chlorophyll metabolism	2/79	0.56871	0.019231
Sphingolipid metabolism	2/89	0.60199	0.035088
Glycolysis or Gluconeogenesis	2/90	0.62074	0.083333
Aminoacyl-tRNA biosynthesis	3/194	0.69254	0.013699
Fatty acid elongation	2/98	0.69748	0.027027

To validate the transcriptomic and metabolomic results, HBE cells were divided into four groups: p53-wt, p53-wt treated with SiO_2 _(p53-wt-SiO_2_), p53-KO, and p53-KO cells treated with SiO_2_ (p53-KO-SiO_2_). Cells were harvested 24 h after 12.5 µg/L, and the IL4I1, HPD, ALDH3A1, ACLY, ACO, ALOX15, CYP2J2, and CYP1A2expression levels were determined using qRT-PCR. As shown in Fig. 5c, compared with the untreated p53-wt group, theIL4I1, HPD, ALDH3A1, ACLY, and ACO expression levels were significantly increased, but the ALOX15, CYP2J2, and CYP1A2 expression levels were significantly reduced in p53-wt-SiO_2_. On the other hand, compared with untreated p53-KO cells, IL4I1, HPD, ALDH3A1, ACLY, and ACO expression levels were significantly reduced, whereas ALOX15, CYP2J2, and CYP1A2 expression levels were significantly reduced in the p53-KO-SiO_2_ cells.

### Benzeneacetic acid is a p53-dependent metabolite that regulates EMT in HBE cells in response to silica

**Fig. 6:**
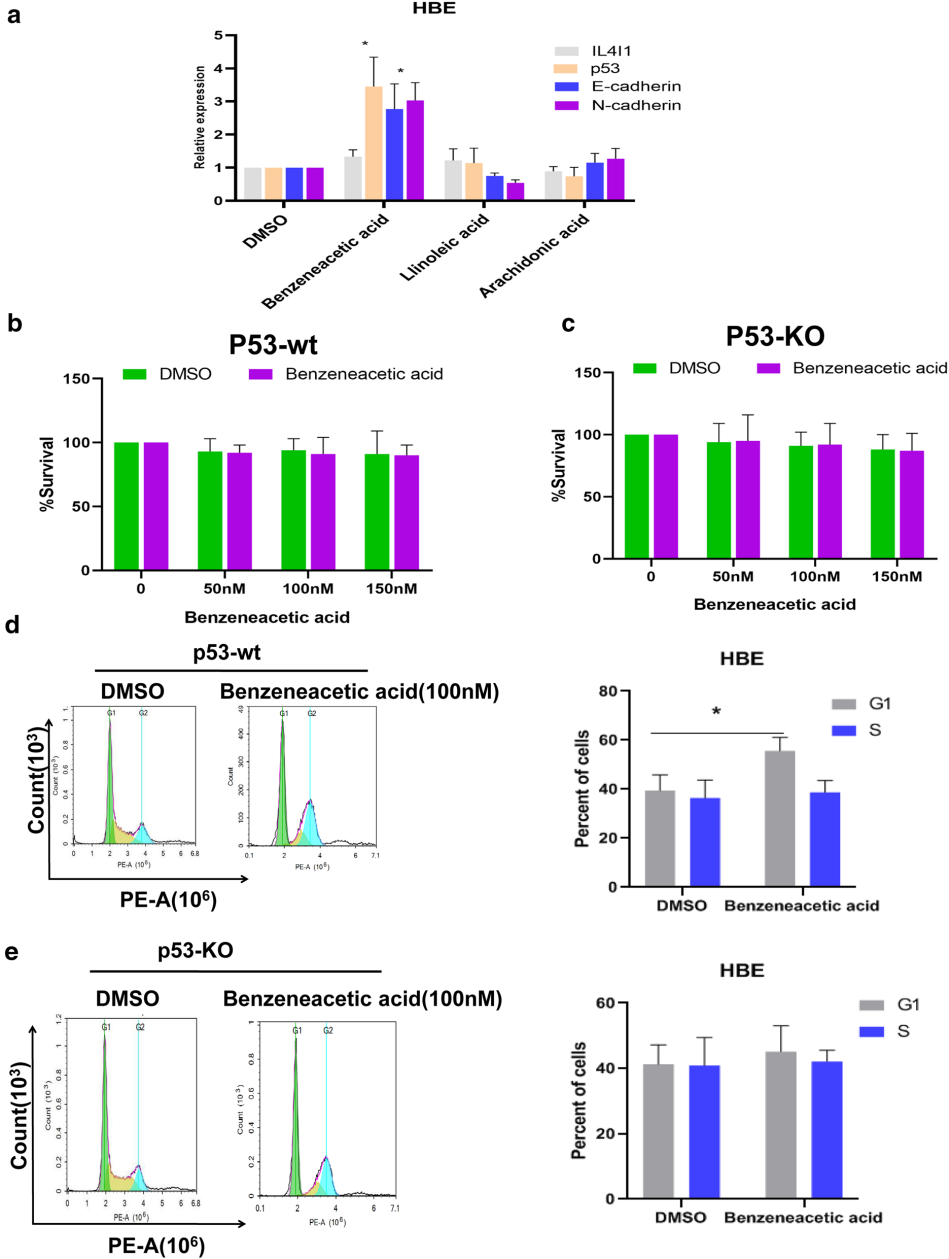
Benzeneacetic acid attenuated silica-induced EMT progression in a p53-dependent manner. **a** Effects of benzeneacetic acid, linoleic acid, and arachidonic acid (concentration = 100 nM) on relative gene expression of IL4I1, p53, and E-Cadherin in HBE cells. **b** Effects of benzeneacetic acid on p53-wt HBE cell survival rate (%) at indicated concentrations (50 nM, 100 nM, 150 nM). **c** Effects of benzeneacetic acid on p53-KO HBE cell survival rate (%) at indicated concentrations (50 nM, 100 nM, 150 nM). **d** Effects of benzeneacetic acid (concentration = 100 nM) on cell cycle in HBE cells. **e** Effects of benzeneacetic acid (concentration = 100 nM) on cell cycle in HBE cells with p53-KO

**Fig. 7:**
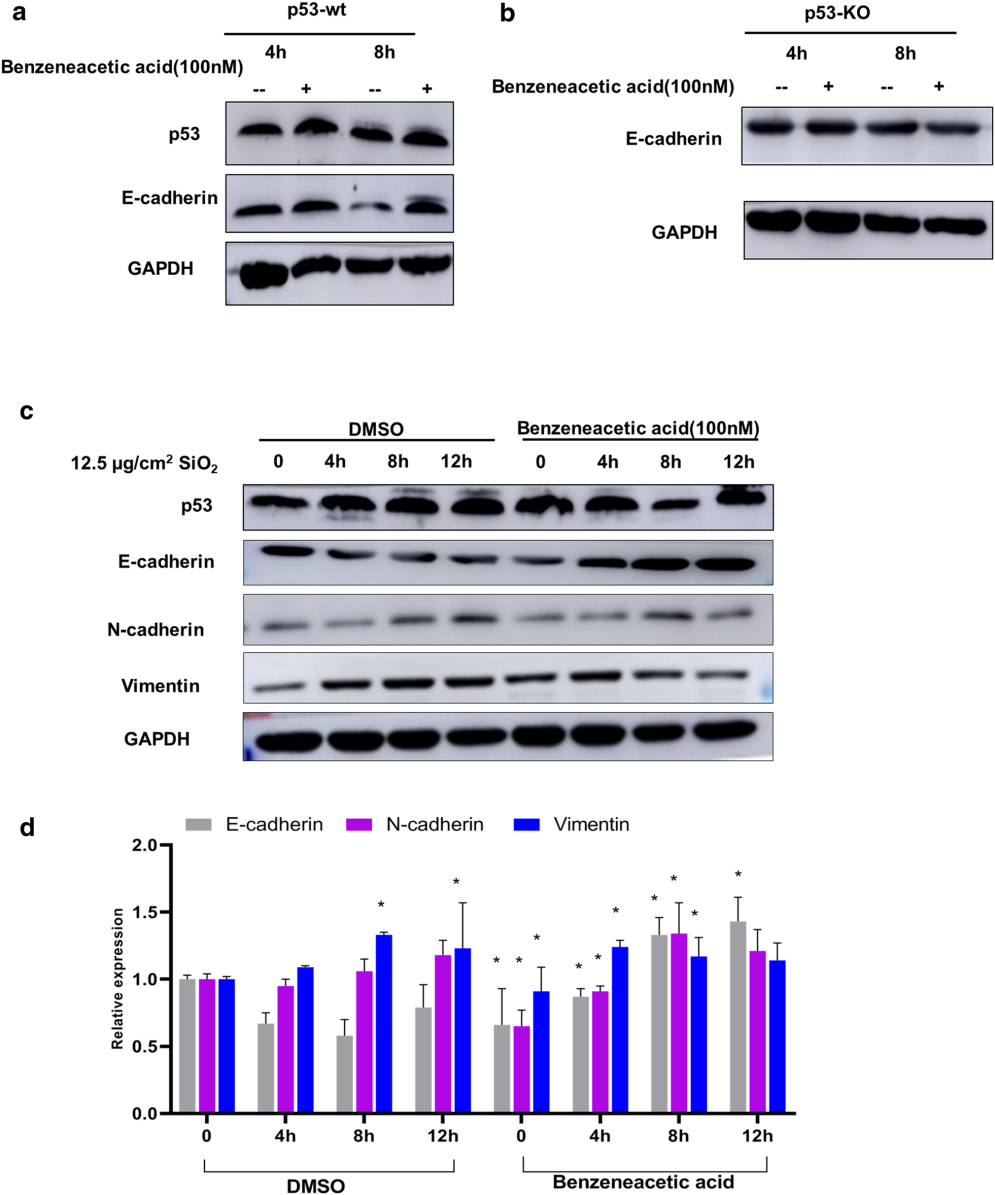
**a** Effects of benzeneacetic acid (concentration = 100 nM) on p53, E-Cadherin expression at indicated time points in p53-wt HBE cells. GAPDH was used as the control. **b** Effects of benzeneacetic acid (concentration = 100 nM) on p53, E-Cadherin expression at indicated time points in p53-KO HBE cells. **c** Effects of benzeneacetic acid (concentration = 100 nM) on the expression of p53, E-Cadherin, N-cadherin, Vimentin at indicated time points in p53-wt HBE cells post 12.5 µg/cm^2^ silica treatment. **d** Quantitative determination of relative E-Cadherin, N-cadherin, Vimentin expression in HBE p53-wt treated with or without benzeneacetic acid exposure at the indicated time points. GAPDH was used as control. Data are presented as means ± SD from three independent experiments: * *p* < 0.05 between different groups

Based on the integrative data provided above, we wondered whether there are key metabolites of p53-dependentinvolvement in the silica-induced EMT progression. We first conducted qRT-PCR to test several inflammation and EMT-related changes in genetic expression such as thatofIL4IL, p53, and E-cadherin treated with or without a few metabolites including benzeneacetic acid, linoleic acid, and arachidonic acid. As shown in Fig. 6a, compared with the DMSO control group, HBE cells treated with 100 µM benzeneacetic acid showing significantly increased levels of p53 and E-Cadherin whereas linoleic acid and arachidonic acid had no significant effects on IL4IL, p53 and E-Cadherin expression. Additional file 3: Fig. S3A show the E-cadherin through immunofluorescence (IF) expression in HBE cells treated with silica, while Additional file 3: Fig. S3B show the actin fiber formation in HBE cells treated with silica. As shown in Fig. 6b, HBE cells treated with benzeneacetic acid for 48 h at indicated concentration showed no significant cell toxicity through CCK-8 detection at the indicated doses, whereas in HBE p53-KO cells, benzeneacetic acid for 48 h at indicated concentration showed no significant cell toxicity (Fig. 6c). We also observed that p53-wt cells treated with benzeneacetic acid led to G1/S arrest, whereas in p53-KO cells, the G1/S transition was not affected by benzeneacetic acid treatment. This suggested that the G1/S arrest activation was p53-dependent (Fig. 6d, e). This result also is in line with previous study that benzeneacetic acid could be an inducer of G0/G1 arrest without significant cell toxicity [34–36]. Similarly, in p53-wt cells treated with benzeneacetic acid, p53 and E-Cadherin expression increased at the indicated time points, whereas in p53-KO cells, E-Cadherin expression was not significantly altered (Fig. 7a, b). Additional file 4: Fig. S4A-B shows the quantitative determination of relative p53 and E-cadherin expression in HBE p53-wt and HBE p53-KO cells post benzeneacetic acid exposure at the indicated time points. As of our previous published study have demonstrated that silica can induce EMT in HBE cells with p53 wild-type expression. Further, a deficiency of p53 led to decreased E-cadherin expression and increased N-cadherin andα-SMA expression in the HBE cells treated with silica, indicating the p53 plays critical role in the regulation of development of EMT [37]. Moreover, we performed other EMT biomarkers including N-cadherin, Vimentin, IL4I1 protein expression in HBE cells with or without p53 knockout post silica exposure (Additional file 5: Fig. S5).

We further used silica (12.5 µg/cm^2^) to treat p53-wt cells and detected changes in EMT biomarker proteins at indicated time points with or without intervention with benzeneacetic acid. The results showed that benzeneacetic acid increased p53 and E-Cadherin expression and decreased N-cadherin and Vimentin expression (Fig. 7c), suggesting that intervention with benzeneacetic acid may attenuate the silica-induced EMT progression.

In summary, these results indicated that benzeneacetic acid may be a potential target for silica induced EMT. These findings are consistent with the transcriptomic and metabolomic results, providing further evidence that the benzeneacetic acid in the dysregulation of silica-related metabolic pathways may be p53-dependent. They also support the importance of p53 in the regulation of silica-induced cell EMT.

### p53-related research trends revealed the status of few transformations

**Fig. 8:**
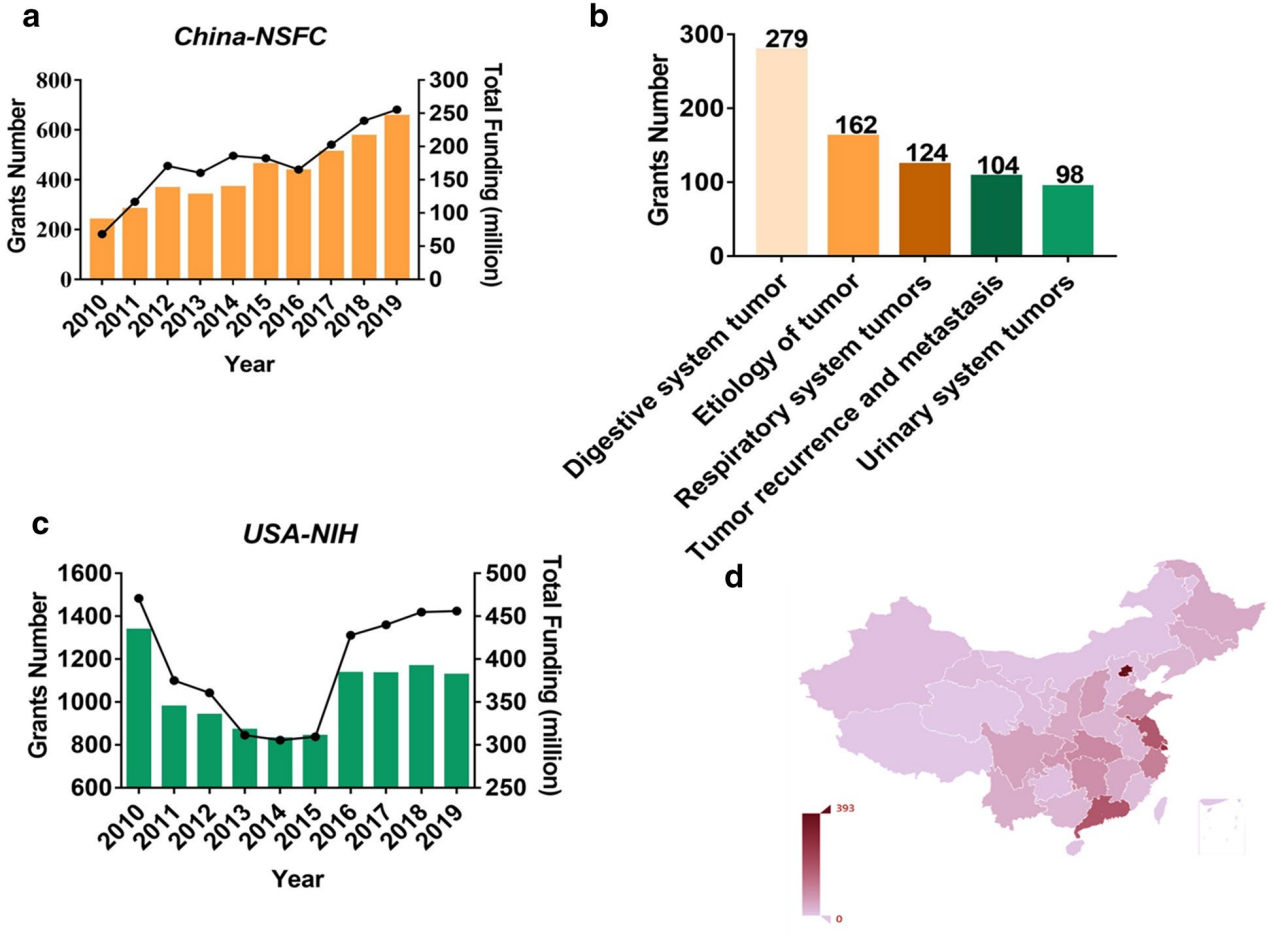
p53 research trends in the past decade. **a** Funding trends and rewarded amounts (RMB, million) in the past three decades by NSFC, China. **b** Percentage of differential fields of funding from NSFC for p53 during 2010–2019. **c** Funding trends and rewarded amounts ($, million) in the past three decades by NIH, USA. **d** Visual presentation of the funds across provinces

Since its discovery, p53 research-related publications have increased substantially. NSFC is the main source of investment for studies on p53 in China. As illustrated in Fig. 8a, from 2010 to 2019, 4225 projects were founded and the total amount of RMB allocated was approximately 1600 million. The funding amounts of the grants and numbers increased every year, with a slight decrease in 2016. The funded project content was mainly comprised of tumor etiology, digestive, respiratory, and urinary system tumors, and tumor recurrence and metastasis. Furthermore, to provide comparative information for the USA, we extracted p53-related research data from the NIH website. Between 1910 and 2019, the NIH funded a total of approximately 10,800 projects relating to p53 research, with an approximate funding amount of USD 7200 million, substantially greater than that of the NSFC (Fig. 8b, c). To explore the institutions funded by NSFC, we used ArcGIS software to present the information visually. As shown in Fig. 8d, the funded projects were concentrated in developed areas or high-income provinces such as Beijing, Shanghai, and Guangdong Province.

**Fig. 9:**
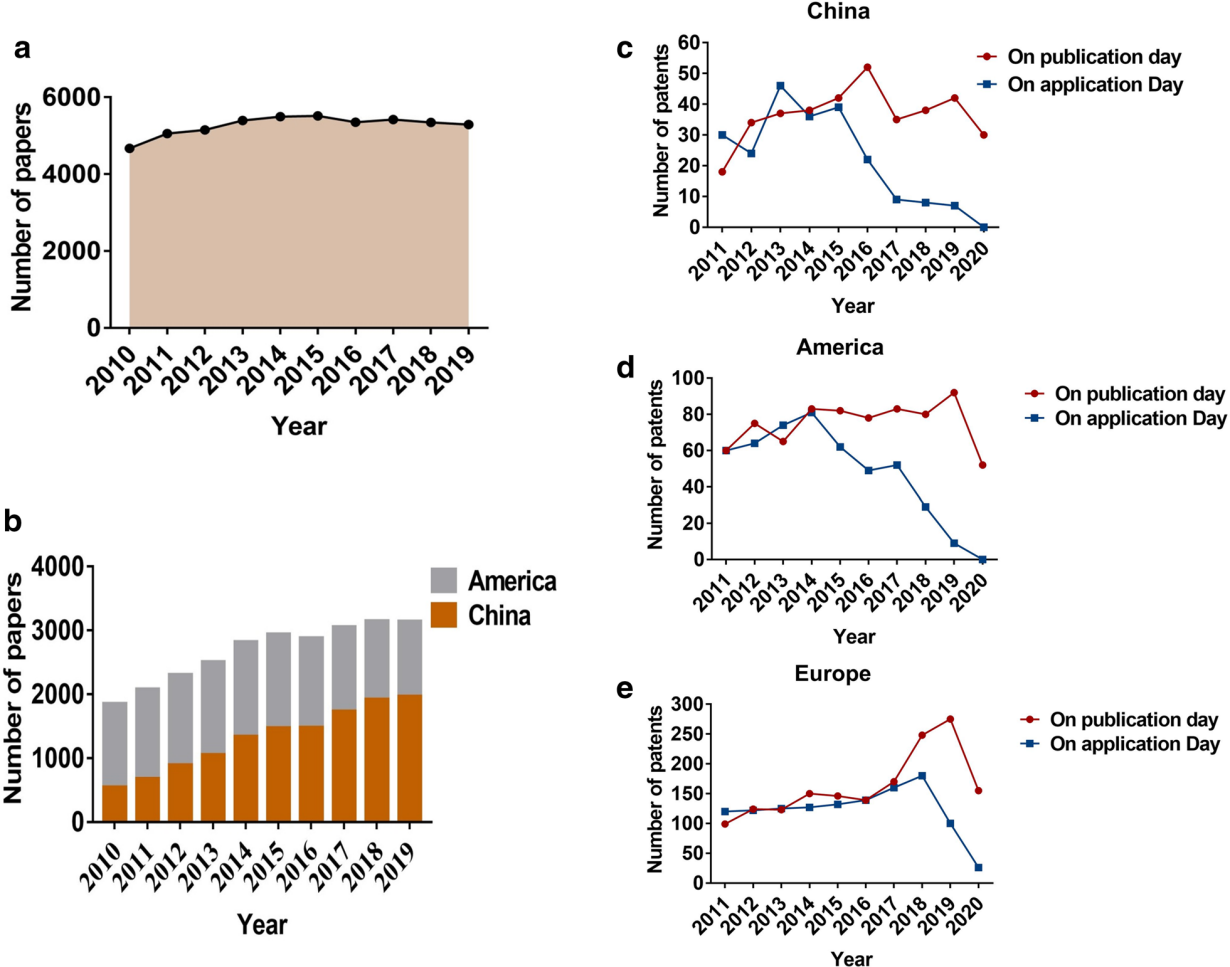
p53 publications in the past decade. **a** The number of p53 studies from 2010 to 2019. **b** Comparative presentation of p53 studies between China and the USA. **c** Awarded invention patents across China, the USA, and Europe from 2011 to 2020

As illustrated in Fig. 9a, from 2010 to 2019, the annual change was not large and remained stable in the 4000 to 5000 studies annually worldwide. However, compared with the USA, Chinese scholars have contributed increasingly to scientific findings. An interesting thing is that the number of publications from Chinese researchers has increased much more rapidly than that in the USA from 2017 onward, indicating that p53 research has been focused on in China (Fig. 9b). We further investigated the awarded invention patent information among China, America, and Europe. From 2011 to 2020, there are only approximately 30–50 annual awarded p53-related invention patents in China. The number of US invention patents was between and 20–100 annually from 2011 to 2010, however, the numbers were greater in Europe compared with China and USA. This may be because Europe includes many developed countries such as Germany. However, in these three regions, from 2017, the number of invention patents decreased significantly (Fig. 9c, d). The p53 research trends analysis revealed that although a large number of p53-related studies have been published, the translation from the bench to the bedside has been stagnant.

## Discussion

Beyond p53 inactivation is essential for majority of human tumors, broading our view of p53 role in silicosis will extremely aid understanding as to how a disease caused by silica, the ubiquitous airborne contaminant can lead to EMT progress. p53 is one of the most extensively studied cancer and environment stress-related adaptive proteins in the past four decades [38]. Strategies that target mutant p53 expression, focusing on degradation of mutated protein, restoring the wild type activity, forming p53 complexes with other proteins, or interfering with p53-related signaling pathways can potentially help prevent and control cancer [14, 38]. However, many related studies remain controversial. For instance, in lung cancer cells, p53 attenuated glucose uptake leading to glycosis inhibition, whereas, in muscle cells, p53 can induce glycolytic enzymes. The possible reason mainly depends on the research context which was pointed out by Edward et al. that the disparate p53activities and functions can be interpreted in different contexts [14]. Hence, in this study, we tried to knockout p53-wt from HBE cells and investigated the resulting interference in the downstream metabolic pathways. Our study revealed that following silica exposure resulted in HBE cell dysfunction through52 mRNAs that were putatively involved in p53-dependent pathways. Metabolomic profiling and bioinformatics analysis revealed that 42 metabolites were putatively involved in p53-dependent silica-mediated HBE cell dysfunction. Through integrated data analysis, we obtained three significant p53-dependent metabolic pathways, including phenylalanine, glyoxylate, dicarboxylate, and linoleic acid metabolism, and the TCA. In particular, we identified roles for benzeneacetic acid, a key regulation metabolite in the phenylalanine metabolic pathway, attenuated the silica-induced EMT in HBE cells in a p53-dependent manner.

Evidence shows that normal epithelial cells may be involved in controlling EMT in complicated mechanisms [39]. The classical function of p53 is to be a transcription cofactor for cells in response to various environmental stresses, including cell cycle arrest, apoptosis, and senescence [40]. However, recent findings suggest that p53 restricts epithelial cell plasticity [41]. This partly occurs due to the initiation of the p53-MDM2-SLUG pathway to enhance the epithelial biomarker E-Cadherin expression [42]. p53 also negatively regulates EMT progression by enhancing miR-34a expression to inhibit its downstream Snail1 expression, a mesenchymal marker that induces EMT progression [43]. Additionally, p53 is expressed in mesenchymal cells, indicating that there may be additional molecular mechanisms involved in the maintenance of epithelial integrity. In normal epithelial cells, p53 protein expression is augmented upon different stresses, including DNA damage and hyperproliferative signals [39]. p53 is also involved in the regulation of silica induced EMT progression. Cho et al. indicated that activated TβR1 by silica induces the phosphorylation and degradation of RKIP, consequently resulting in Snail-mediated p53 suppression and the occurrence of EMT [44]. In this study, we compared the mRNA alterations and found that ANKRD22, GALNT16, LGR5, ITGA9, ELMOD1, SLC9B1, GOLGA8Q, FAM19A5, PLA2G2C, and LAIR2 were significantly changed post silica exposure when p53 was deficient. In p53-KO murine gastric epithelial (GIF-14) cells, EMT-induced plasticity is reflected in the expression of the embryonal proto-oncogene LGR5 [45], whereas the relationship between p53 and other mRNAs in EMT progression has not been reported. Further studies are warranted to confirm the alternations of these mRNAs in silica-induced p53 deficiency.

p53 regulation may be involved in EMT-related metabolic pathways. Sabine et al. suggested that treatment with calcitriol, the active vitamin D3 metabolite, activates p53 expression, and increases the repression of SNAIL, inhibiting EMT progression [46]. Fu et al. showed that restriction of phenylalanine modulates p53 and glucose metabolism in prostate cancer cells [47]. Karin et al. indicated that phenylalanine hydroxylase (PAH) is involved in the regulation of tyrosinase via p53 through the transcription of hepatocyte nuclear factor 1 alpha, which regulates melanogenesis [48]. Indeed, p53 controls multiple metabolic pathways, including glycolysis and pentose phosphate pathway, lipid, sphingolipid, amino acid, ammonia, and iron metabolism, mitochondrial biogenesis, integrity and respiration, TCA cycle, and ferroptosis [49] in normal cells, suggesting that deregulation of any associated metabolic activities contributes to several cancer and non-cancer human pathologies. Through this, it is obvious that p53 functions in metabolic pathways and displays certain cell and tissue specificity [49]. Thus, despite the classical function of the “guardian of the genome”, p53 may be better characterized as a “guardian of homeostasis”. Here, we report that phenylalanine, glyoxylate, dicarboxylate, and linoleic acid metabolism, and TCA may be involved in silica-stimuli in a p53-dependent manner. Phenylalanine metabolism belongs to amino acid metabolism. A recent study demonstrated that the retention of l-arginine, a semi exponential amino acid, increased p53 expression, and reduced the contents of pro-inflammatory cytokines in hepatic cells [50]. For TCA, the onco-metabolites stabilize the hypoxia inducible factor 1, inhibit p53 expression, and increase glutaminolysis, glycolysis, or dysregulation of EMT, suggesting that these changes in p53 and TCA-related metabolites are important driving forces of cancer pathogenesis and progression [51]. However, few studies have been reported on the p53-dependent changes in relative metabolites following silica exposure. We used integrative multiple-omics analysis to provide baseline information for further p53 studies.

Benzeneacetic acid, was one of the major compounds among the 31 substances found in the ethyl acetate extract [52]. A previous study has shown that paraquat exposure in rats increases benzeneacetic acid levels [53]. Benzeneacetic acid has also been considered as an anti-inflammatory compound with the function of inhibiting lipopolysaccharide-induced inflammation in vivo [54]. Asynchronized smooth muscle cells treated with S-diclofenac (2-[(2,6-dichlorophenyl) amino] benzene acetic acid 4-(3H-1,2,dithiol-3-thione-5-yl) phenyl ester) showed an increase in stabilization of p53 [55]. In the KEGG database, benzeneacetic acid is involved in phenylalanine metabolism, and ALDH3, feaB, and paaI are thought to promote its levels, thereby increasing phenyl-acetylglutamine. Despite these reports, a mechanistic link between benzeneacetic acid and silica induced EMT has not yet been reported. The present study confirms the major role of p53-dependent benzeneacetic acid generation in normal HBEs post the silica stimulus. Notably, increased benzeneacetic acid levels in p53-wt cells inhibited the silica-induced EMT progression. The present study suggests that certain clinical benefits of benzeneacetic acid observed with p53 existence status may be in part mediated via attenuation in silica-triggered EMT signaling events in vitro. Although the present study focuses only on the function of benzeneacetic acid in human HBE cells, one can speculate that benzeneacetic acid has the potential to modulate other EMT-related markers or pathways, and accordingly, may potentially influence multiple silica-linked physiological processes.

Research trends regarding p53 research and control provide a closer look at this field. Since its discovery 40 years ago, p53 remains heavily investigated in the global scientific community, specifically in the USA. China has rapidly advanced the understanding of p53 in recent years, with large government investments through NSFC foundations. However, the clinical translation of p53 research is still challenging, despite a few studies that have applied for invention patents. This reflects the lack of innovative research and innovation. Olivier et al. emphasized that p53 translational research for improving cancer detection, prognosis, prevention, and therapy based on p53 study is critical in qualitative terms. p53 research has been conducted for three periods: the discovery from 1980 to 1990, first considered an oncogene factor; the rapid development study period from 1990 to 2010, second mainly characterized as multi-functional (transcription factor and causing double-edge effects in the various contexts); the third period was from 2010 to present, where research lacks substantial findings and clinical translation has been stagnating. At this time, we should encourage and incentivize high-impact metabolic future studies that use rare human disease contexts and novel animal models that can be clinically translatable.

Although p53 research has been intensive and obtained extremely discoveries, there still remains much to explore for p53 origin and regulation. Challenge in the coming era of p53 research would be the first of the comprehensive p53 function in other diseases instead of cancers, the second is the urgent need to translate p53 research results into clinical application. And new technologies applied in p53 function explore is also a big challenge for more robust clinical advances.

##  Supplementary Information

**Additional file 1: Fig. S1.** Top 30 differential expressed genes’ GO assay in p53-wt and p53-Ko cells, respectively.

**Additional file 2: Fig. S2.** Verification of differential expressed genes in p53-wt and p53-Ko cells, respectively.

**Additional file 3: Fig. S3.**(A) IF illustration of E-cadherin in HBE cells treated with silica. (B) actin fiber formation in HBE cells treated with silica.

**Additional file 4: Fig. S4.** (A) Quantitative determination of relative p53 expression in HBE p53-wt post benzeneacetic acid exposure. (B) Quantitative determination of relative E-cadherin expression in HBE p53-wt and HBE p53-KO cells post benzeneacetic acid exposure at the indicated time points.

**Additional file 5: Fig. S5.** Expression of E-cadherin, N-cadherin, Vimentin and IL4I1 in HBE p53-wt and HBE p53-KO cells post benzeneacetic acid exposure at the indicated time points.

## Data Availability

Not applicable.
