# Supplementary figures and images for: Transcriptomic and metabolomic profiling reveal the p53-dependent benzeneacetic acid attenuation of silica‐induced epithelial–mesenchymal transition in human bronchial epithelial cells
Source: Cell Biosci. 2021 Feb 5;11:30. doi: 10.1186/s13578-021-00545-0 (PMC7866764; doi:10.1186/s13578-021-00545-0)

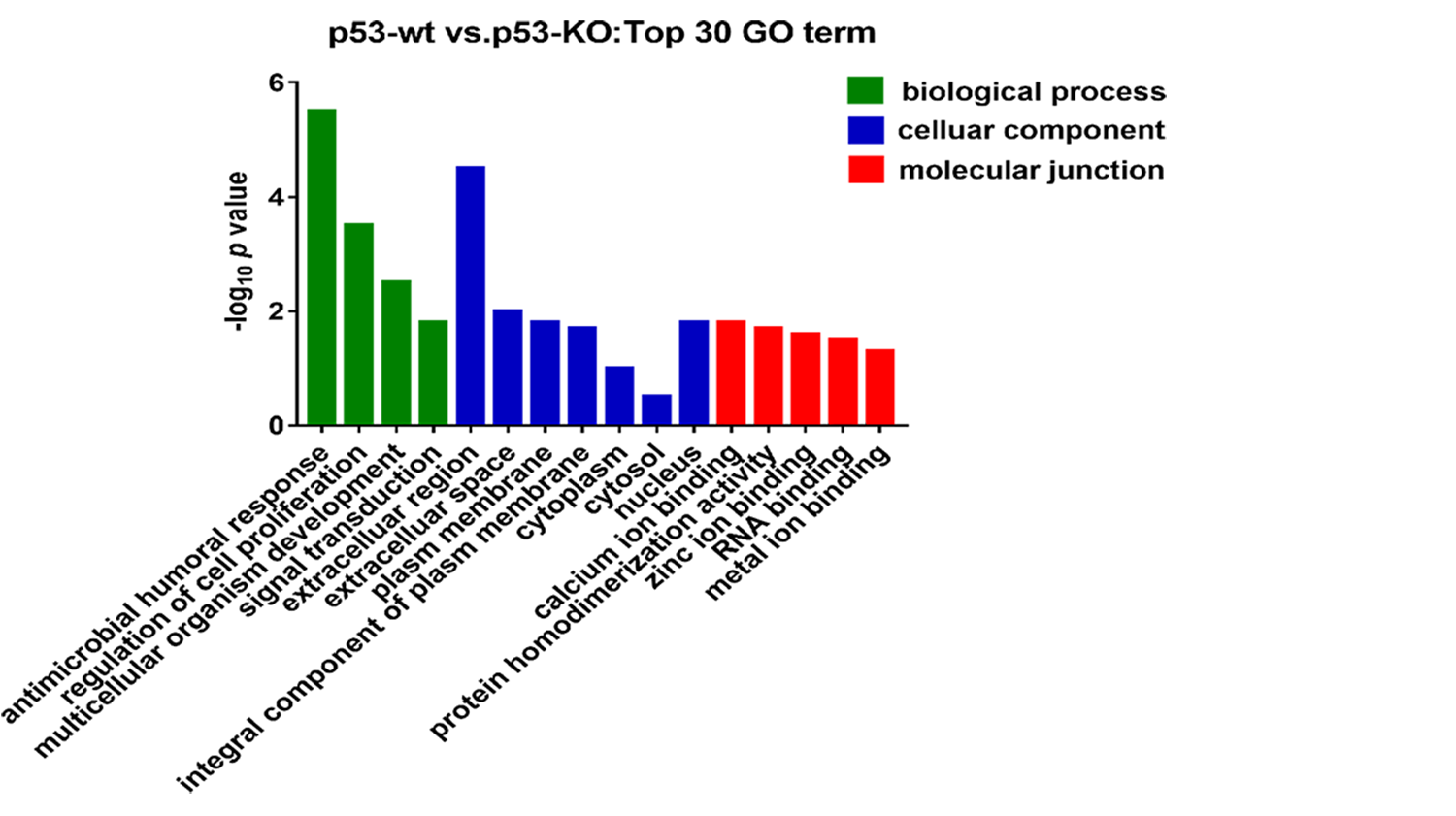

Supplement: Supplementary file 1 — Additional file 1: Fig. S1. Top 30 differential expressed genes’ GO assay in p53-wt and p53-Ko cells, respectively. [file 13578_2021_545_MOESM1_ESM.tif]

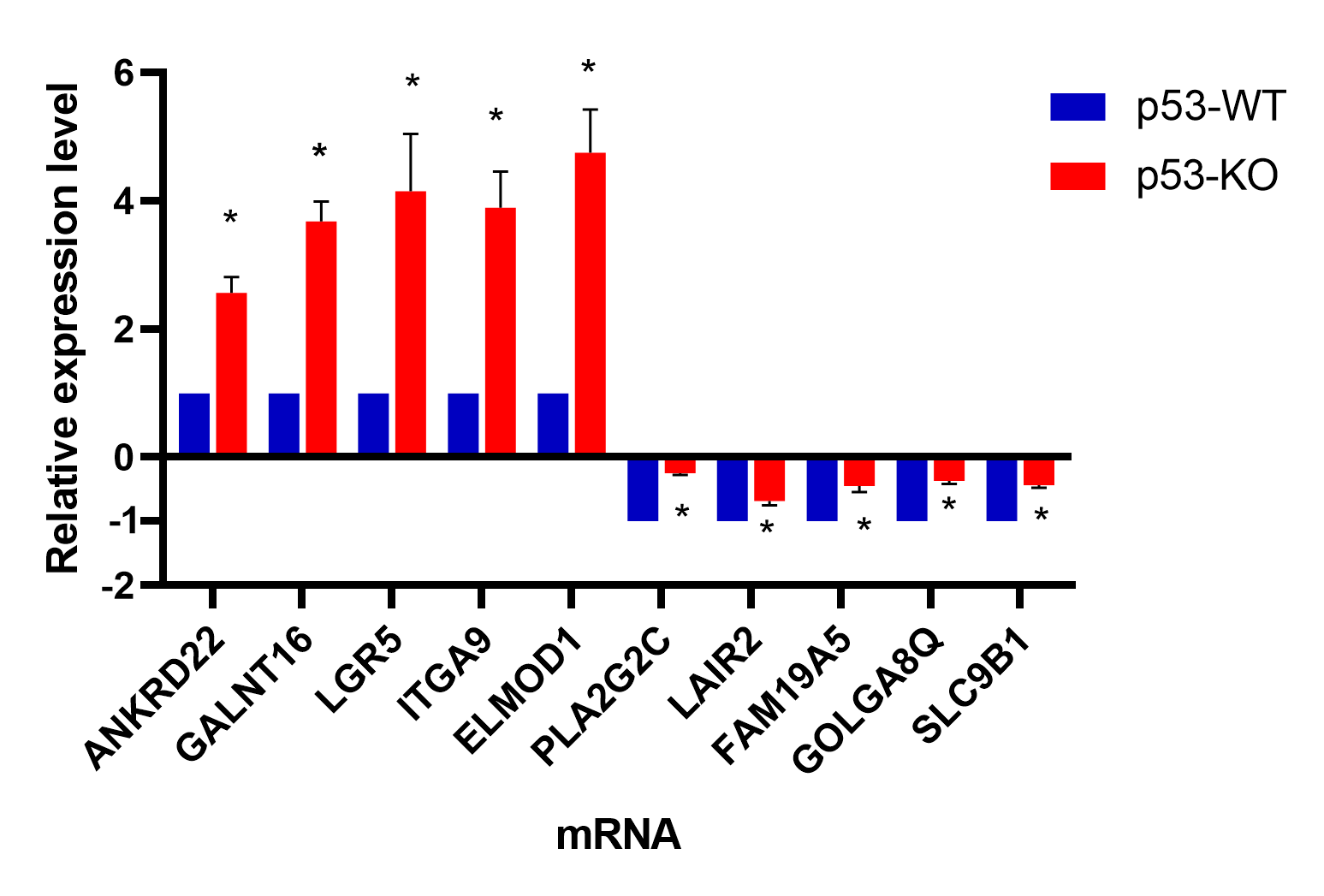

Supplement: Supplementary file 2 — Additional file 2: Fig. S2. Verification of differential expressed genes in p53-wt and p53-Ko cells, respectively. [file 13578_2021_545_MOESM2_ESM.tif]

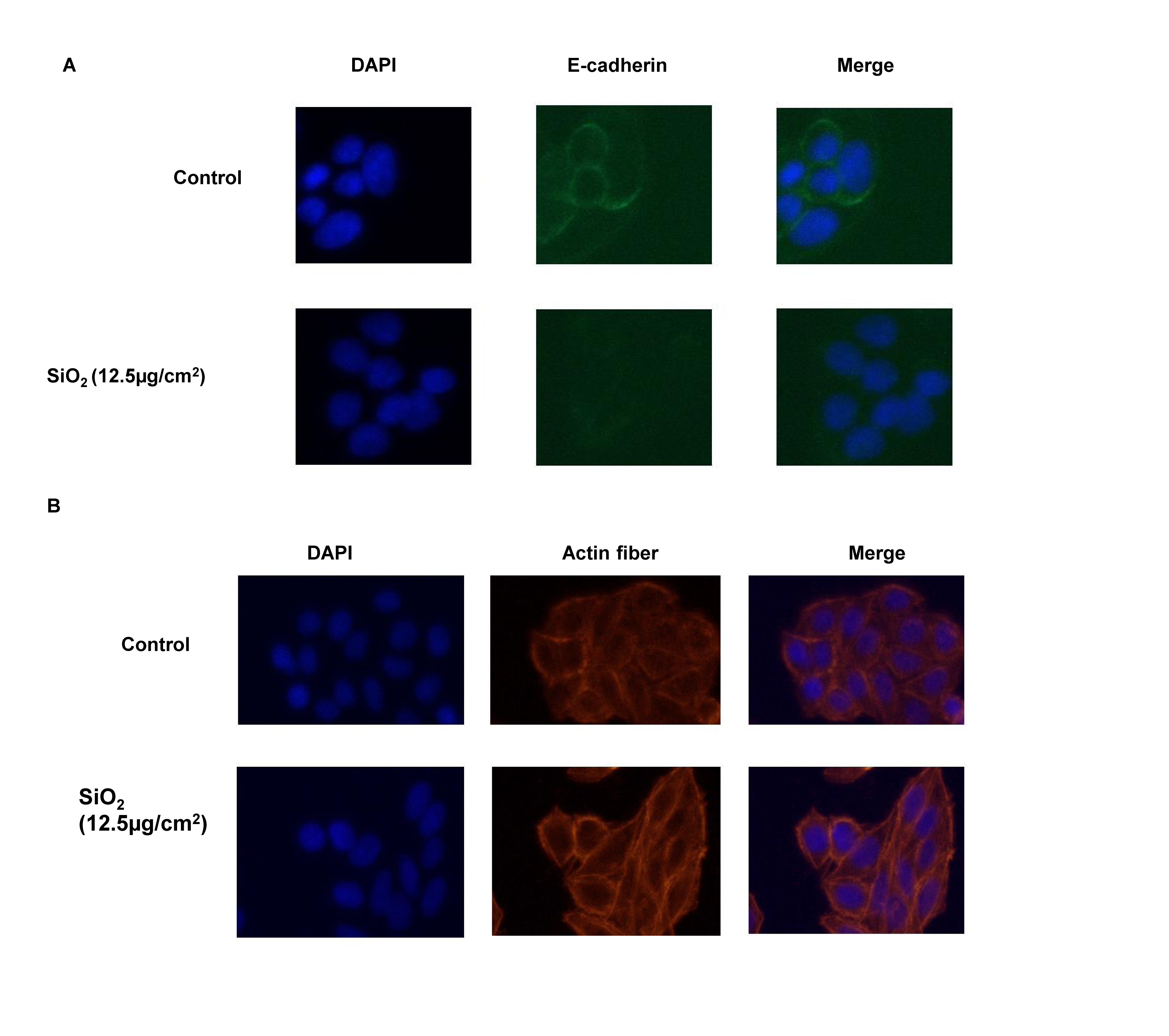

Supplement: Supplementary file 3 — Additional file 3: Fig. S3.(A) IF illustration of E-cadherin in HBE cells treated with silica. (B) actin fiber formation in HBE cells treated with silica. [file 13578_2021_545_MOESM3_ESM.tif]

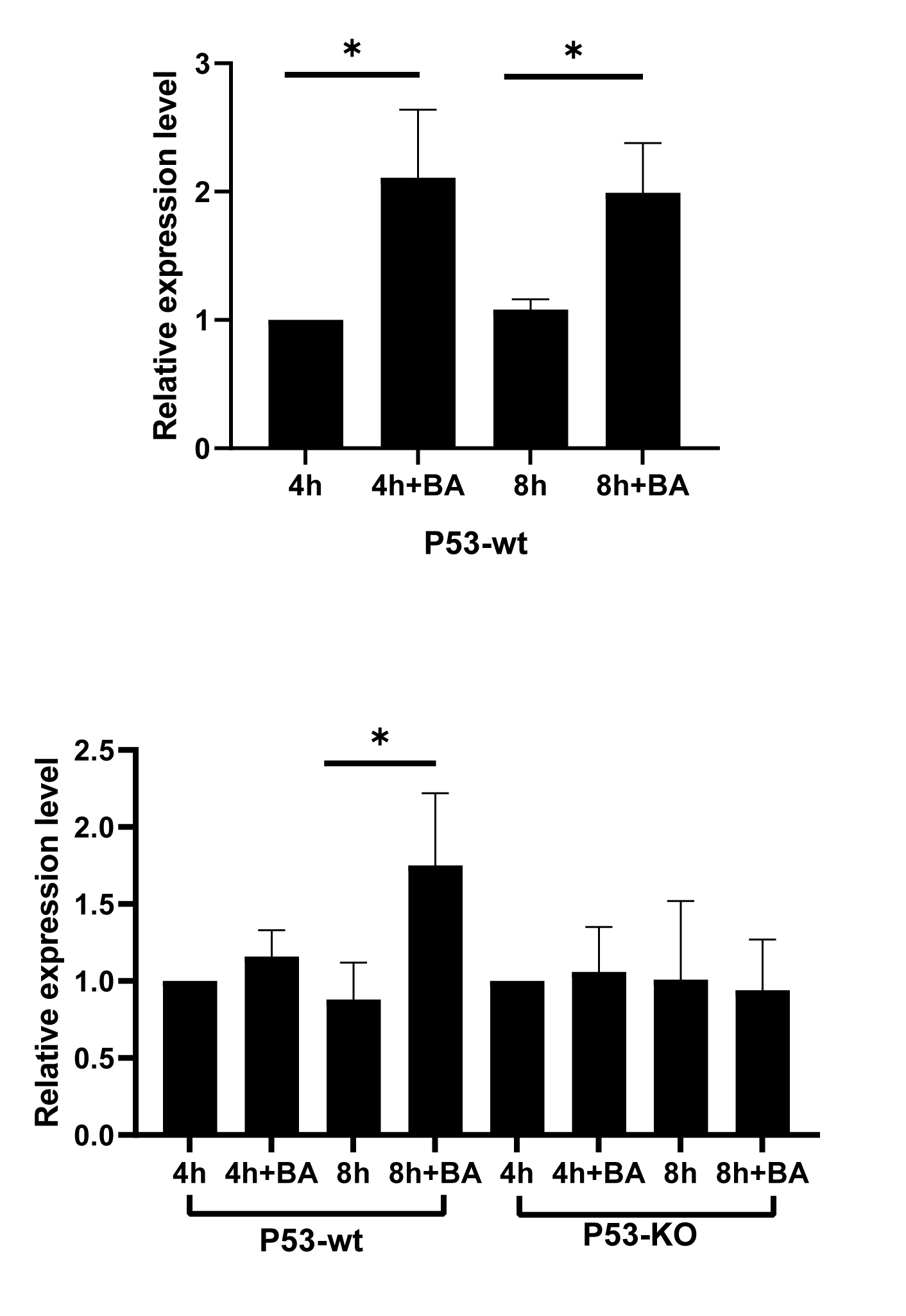

Supplement: Supplementary file 4 — Additional file 4: Fig. S4. (A) Quantitative determination of relative p53 expression in HBE p53-wt post benzeneacetic acid exposure. (B) Quantitative determination of relative E-cadherin expression in HBE p53-wt and HBE p53-KO cells post benzeneacetic acid exposure at the indicated time points. [file 13578_2021_545_MOESM4_ESM.tif]

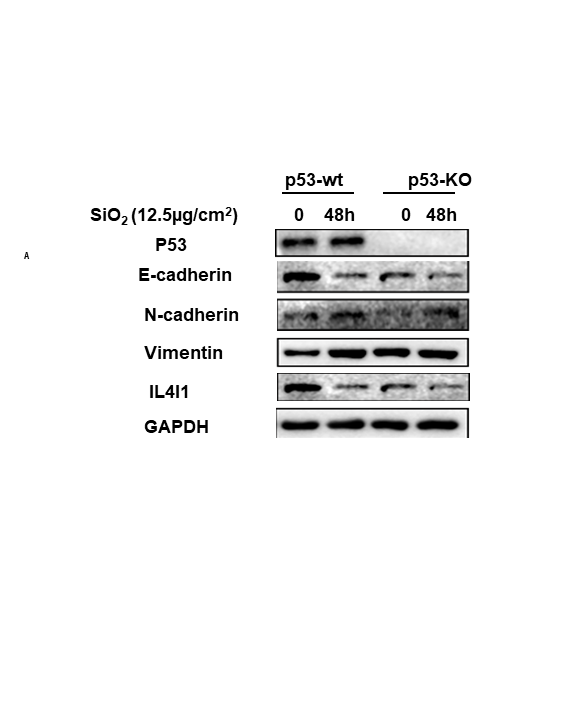

Supplement: Supplementary file 5 — Additional file 5: Fig. S5. Expression of E-cadherin, N-cadherin, Vimentin and IL4I1 in HBE p53-wt and HBE p53-KO cells post benzeneacetic acid exposure at the indicated time points. [file 13578_2021_545_MOESM5_ESM.tif]
